# Nanobubble-Induced Oxygen Gradients Control Corrosion Mode and Bacterial Adhesion on Martensitic Stainless Steel

**DOI:** 10.1021/acsomega.6c09261

**Published:** 2026-09-29

**Authors:** Kacper Kołodziejski, Maja Goworek, Karol Ulatowski, Rafał Podgórski, Paweł Sobieszuk

**Affiliations:** 49566 Warsaw University of Technology, Faculty of Chemical and Process Engineering, Department of Biotechnology and Bioprocess Engineering, Waryńskiego 1, Warsaw 00-645, Poland

## Abstract

Biofilm formation is a large problem in corrosion prevention and can be part of a positive feedback loop, where a corroded surface is prone to bacterial adhesion, and bacteria themselves provide an environment that increases the corrosion rate of the metal surface. Nanobubbles are increasingly recognized as active modifiers of solid–liquid interfaces; however, their role in coupled corrosion and biofilm formation remains unclear. Here, we show that gas-specific nanobubble dispersions control both the corrosion mode of martensitic stainless steel (4H13) and the resulting *Escherichia coli* adhesion. Steel samples were immersed in oxygen or nitrogen nanobubble dispersions under varying salinity conditions up to 1.0 M NaCl. Oxygen nanobubbles induced spatially heterogeneous corrosion, characterized by localized pitting and differences along the sample height, while nitrogen nanobubbles resulted in uniform surface degradation, even in deionized water. These corrosion patterns directly translated into distinct biofilm structures: patchy biofilms formed on oxygen-treated surfaces, whereas nitrogen nanobubbles promoted a uniform biofilm. These observations are consistent with nanobubble-driven oxygen transport at the interface. We propose that an oxygen concentration gradient along the length of the steel coupon promotes differential aeration cells and pitting corrosion, whereas nitrogen nanobubbles deoxygenate the system, destabilizing the passive layer and leading to uniform corrosion. Consistent qualitative observations from both crystal-violet and DAPI staining reveal a strong coupling between nanobubble-mediated oxygen gradients, corrosion morphology, and microbial colonization. These findings highlight nanobubbles as a simple tool for controlling interfacial electrochemical processes and biofilm formation.

## Graphical abstract

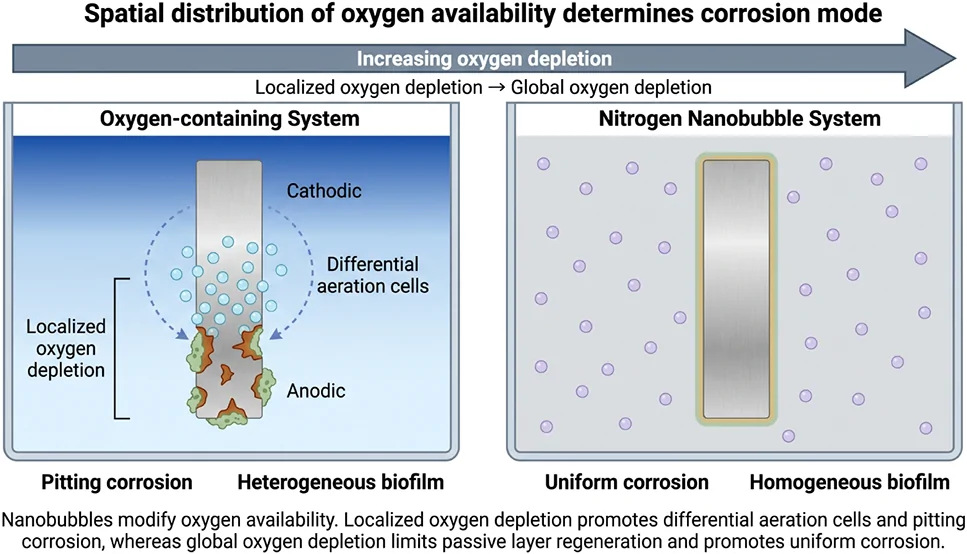

## 1 Introduction

Corrosion of steel components in contact with aqueous environments remains a major global challenge, accounting for losses estimated at approximately 3.4% of global GDP.^1^ A significant fraction of global steel production is used to replace corroded infrastructure and devices.^2^ This issue is also relevant to nanobubble generation systems, which are commonly constructed from stainless steel and operate under prolonged contact with water.

Electrochemical corrosion of steel is governed by the formation of anodic and cathodic regions on the metal surface, typically driven by local differences in oxygen concentration.^3,4^ Spatial heterogeneity in dissolved oxygen leads to the formation of differential aeration cells, which intensify localized corrosion processes such as pitting.^3,4^ At the same time, corrosion-induced changes in surface morphology, particularly increased roughness and the presence of corrosion products, promote bacterial adhesion and biofilm formation.^5–7^ The presence of biofilm in many cases increases the rate of corrosion, inducing a positive feedback loop.^5^

Nanobubbles, also called ultrafine bubbles,^8,9^ owing to their stability and unique interfacial properties,^10–13^ may significantly modify these processes. In particular, they can influence local oxygen transport,^14^ allow for obtaining higher total gas concentrations at equilibrium with air,^15,16^ reduce the rate of gas concentration change in nonequilibrium states,^13,17^ and interact dynamically with solid surfaces.^18,19^ Depending on the gas type, nanobubbles may either enhance oxygen availability at the interface or limit oxygen transport to the surface.^20,21^ As a result, they have the potential to alter both the corrosion mechanism and the subsequent biological response.^22–24^ However, despite growing interest in nanobubble interactions with solid surfaces,^25–27^ their role in controlling the corrosion mode (e.g., pitting versus uniform corrosion) and its coupling with biofilm formation remains largely unexplored.

We see research on influencing bacterial adhesion to stainless steel by the introduction of nanobubbles as an important aspect in multiple branches of industry and everyday life. For example, biofilm formation and corrosion occur in water taps and armatures, among others. In engineering, bacterial adhesion often occurs on corroded parts of steel used commonly as construction materials, which usually leads to an intensification of corrosion. We wish to find out whether some gases enclosed in nanobubbles may decrease the rate of corrosion, inhibiting bacterial adhesion. On the other hand, the introduction of bacteria onto steel beads for potential use as a solid platform and immobilization site for bacterial growth in bioreactors is often limited by the smoothness of the surface. If one can control the corrosion process to form shallow pores covering a large area of the beads and then stop the corrosion, that would allow for the formation of porous steel beads which will be durable and easy to sterilize.

We hypothesize that nanobubbles regulate oxygen transport along steel surfaces under stagnant conditions, thereby controlling the local electrochemical potentials and corrosion patterns. Here, we demonstrate that oxygen nanobubbles induce spatially heterogeneous corrosion damage of martensitic stainless steel, leading to patchy bacterial adhesion, whereas nitrogen nanobubbles promote uniform corrosion associated with homogeneous biofilm formation. These findings highlight nanobubbles as potential tools for interfacial control of electrochemical and biological processes. To the best of our knowledge, this is the first study linking gas-specific nanobubble dispersions, corrosion mode, and resulting bacterial adhesion within a single experimental framework.

## 2 Materials and Methods

### 2.1 Materials

#### 2.1.1 4H13 Steel

In this investigation, we chose to use the 4H13 martensitic stainless steel (Virgamet, Poland), also known as 1.4034 steel. This type of steel was selected as a model material due to its relatively low corrosion resistance compared to that of austenitic stainless steels, which enables rapid development of observable corrosion features under laboratory conditions. Steel was mechanically cut into 2 cm square samples. Before the experiments, samples were pretreated to remove surface contamination and unify the material’s topography. Pretreatment involved sanding with progressively finer-grained sandpaper and degreasing with water and isopropyl alcohol.

#### 2.1.2 *Escherichia coli* Strain and M9 Medium

The bacterial strain used in this investigation was *E. coli*. It was selected as a model biofilm-forming microorganism due to its well-characterized adhesion behavior and widespread use in surface colonization studies. *E. coli* was cultured in M9 minimal medium (DSMZ Medium 382) prepared according to the protocol from the Leibniz Institute DSMZ-German Collection of Microorganisms and Cell Cultures. *E. coli* cultures were carried out in Erlenmeyer flasks and then diluted to an OD600 of 0.1.

#### 2.1.3 Corrosive Environment Preparation

The corrosive environment was either ultrapure water obtained from a deionizer (Polwater, Poland) or solutions of NaCl (Chempur, Poland) in ultrapure water to obtain salt concentrations of 0.2, 0.6, or 1.0 M. Three experimental variants were investigated: oxygen nanobubble dispersions, nitrogen nanobubble dispersions, and nanobubble-free controls.

### 2.2 Methods

#### 2.2.1 Nanobubble Generation

Nanobubbles were generated using a setup with a ceramic membrane that was designed by our team in cooperation with the Fine Bubble Technologies Company (Poland). The setup is schematically presented in Figure 1B. The setup was supplied with either nitrogen or oxygen (Air Products, USA) from a cylinder, and the gas was pressurized through the membrane (average pore diameter, 200 nm), where the gas phase was dispersed into the liquid stream by shear forces generated within the membrane module. We used a gas pressure of 1.8 and 1.5 bar of liquid pressure (which corresponded to 5.5 L/min of liquid flow rate). Generation lasted for 20 min. The system generated stable narrow-distribution nanobubbles with an average Sauter diameter of 250 nm for oxygen and 350 nm for nitrogen under the described operation conditions measured by dynamic light scattering using a Zetasizer NanoZS, as shown previously.^28,29^

**Figure 1:**
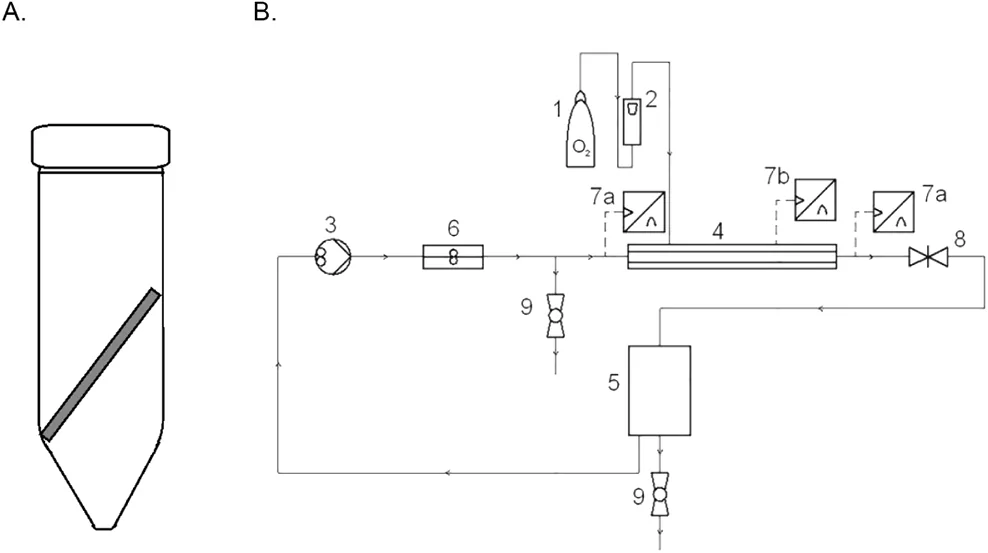
Visualization of sample orientation during corrosion experiments (A) and the nanobubble generation system (B). Nanobubble generation system (Fine Bubble Technologies, Poland): (1) gas cylinder; (2) rotameter; (3) gear pump (Scherzinger, Germany); (4) ceramic membrane module; (5) nanodispersion tank; (6) volumetric flow meter; (7) pressure transducers (UPT20, WIKA, Poland) for liquid (a) or gas (b); (8) gate valve; (9) ball valve.

#### 2.2.2 Corrosion Exposure

After pretreatment (see Section 2.1.1), steel samples were positioned in a skewed manner inside sterile 50 mL Falcon tubes (see Figure 1A) containing freshly prepared corrosive medium. Falcon tubes were filled with corrosive liquid (see Section 2.1.3) to the brim and then covered with a screw cap to reduce the contact of the system with the atmosphere. Samples were stored closed for 7 days and removed from the solutions, flushed with deionized water to remove loose corrosion products, and gently dried using paper towels. All of the experiments were performed in triplicate.

#### 2.2.3 Biofilm Culturing

10 mL of *E. coli* suspension in fresh medium (OD600 equal to 0.1) was poured into 5.5 cm Petri dishes containing steel sample and covered. Petri dishes were then incubated for 72 h at 37 °C without mixing and then the steel samples were removed and thrice flushed with sterile deionized water to remove loosely attached bacteria and air-dried under laminar hood.

#### 2.2.4 Analysis of Biofilm

Quantitative analysis was carried out by using crystal violet dyeing. 1 mL of 0.1% crystal violet in PBS was spread onto steel samples with biofilm and incubated at room temperature for 30 min. Samples were thrice flushed with sterile deionized water and air-dried. Then, samples were destained using 30% acetic acid. The liquid over the samples was then analyzed using spectrophotometry (wavelength, 590 nm).

Qualitative analysis was carried out by using DAPI dyeing. Samples were dyed with 1 mL of 300 nM DAPI dye for 5 min in darkness. Dyed samples were analyzed using confocal microscopy.

## 3 Results and Discussion

### 3.1 Corrosion Patterns and Proposed Oxygen-Driven Mechanism

Before discussing the possible nanobubble impact on the corrosion process, one must be aware of what processes occur there originally. In neutral-pH aqueous solutions, which adhere to our research, as we investigated NaCl solutions, the following electrochemical reactions occur on the surface during corrosion.

Anodic reaction:

1 $${\mathrm{Fe}}_{\left(s\right)}\to {\mathrm{Fe}}_{\left(\mathrm{aq}\right)}^{2+}+2e^{-}$$

Cathodic reaction:

2 $$O_{2\left(g\right)}+2H_{2}O_{\left(l\right)}+4e^{-}\to 4OH_{\left(aq\right)}^{-}$$

When NaCl is present, additional reactions occur:

Near the anode:

$${\mathrm{Fe}}_{\left(\mathrm{aq}\right)}^{2+}+2Cl_{\left(\mathrm{aq}\right)}^{-}\to {\mathrm{FeCl}}_{2\left(\mathrm{aq}\right)}$$

$${\mathrm{FeCl}}_{2\left(\mathrm{aq}\right)}+2H_{2}O_{\left(l\right)}\to Fe{\left(OH\right)}_{2\left(s\right)}+2H_{\left(aq\right)}^{+}+2{\mathrm{Cl}}_{\left(\mathrm{aq}\right)}^{-}$$

Secondary cathodic reaction when pH drops locally due to H^+^ release near the anode:

$$2H_{\left(\mathrm{aq}\right)}^{+}+2e^{-}\to H_{2\left(g\right)}$$

Even though nanobubbles do not introduce additional reagents, they may influence the availability of oxygen due to their high surface-to-volume ratio and number concentration, which will influence the rate of the cathodic reaction.’

Figure 2 presents the macroscopic appearance of 4H13 steel samples after 7 days of exposure to deionized water and NaCl solutions containing oxygen nanobubbles, nitrogen nanobubbles, or no nanobubbles (reference).

**Figure 2:**
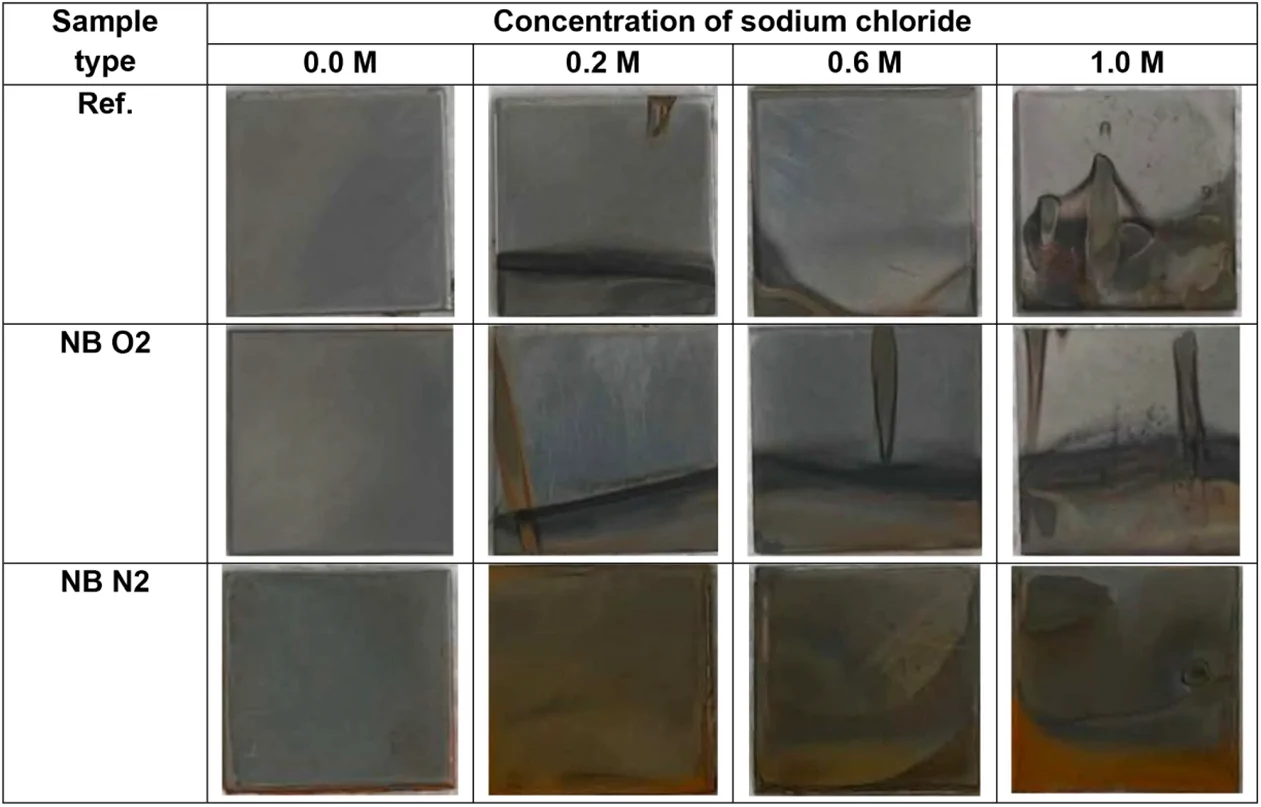
4H13 steel plates after 7-day exposure to the corrosive environment without nanobubbles dispersed in the NaCl solution (denoted as “Ref.”) or with either oxygen nanobubbles (denoted as “NB O2”) or nitrogen nanobubbles (denoted as “NB N2”) dispersed. The black dots are the origins of pitting corrosion, which are also the origins of brownish “leaks” visible, e.g., for 0.6 M NaCl in NB O2. Intensive brown coloration is the signal of the fully developed uniform surface corrosion (see, e.g., 1.0 M in NB N2).

For the reference system, corrosion products were barely visible at low NaCl concentrations, whereas characteristic rust streaks associated with pitting corrosion appeared at higher salt concentrations. A similar corrosion mode was observed for the oxygen nanobubble dispersions. However, visible corrosion developed already at lower salt concentrations, indicating an accelerated degradation of the material. In both cases, corrosion was spatially heterogeneous and was predominantly concentrated in the lower parts of the coupons. In contrast, samples exposed to nitrogen nanobubble dispersions exhibited a markedly different corrosion pattern. Corrosion products were distributed more uniformly across the entire surface, and no pronounced rust streaks originating from localized pits were observed. Interestingly, corrosion was visible even in deionized water containing nitrogen nanobubbles, suggesting a mechanism different from that of conventional chloride-induced pitting corrosion.

The observed corrosion distribution in the reference and oxygen nanobubble systems appears counterintuitive as oxygen nanobubbles could be expected to provide a more homogeneous oxygen supply to the metal surface. A possible explanation is related to the geometry of the experimental systems. Steel coupons were positioned nearly vertically in nearly completely filled Falcon tubes, leaving only a small air pocket beneath the cap. Under such stagnant conditions, dissolved oxygen may gradually be consumed by passivation and corrosion reactions. As oxygen becomes depleted from the bulk liquid, diffusion from the trapped air volume may generate a vertical oxygen concentration gradient. The preferential corrosion of the lower coupon regions is consistent with the formation of differential aeration cells,^1,3,30^ in which oxygen-rich regions act as cathodic areas while oxygen-depleted regions become anodic and corrode preferentially. This interpretation is further supported by the autocatalytic nature of underdeposit corrosion.^30^ Accumulation of corrosion products may locally restrict oxygen transport to the surface, enhancing oxygen concentration differences between intact and corroded regions and promoting a further localized attack.

A distinct explanation is required for nitrogen nanobubble dispersions. During nanobubble generation, nitrogen effectively removes dissolved oxygen from the liquid phase, creating a strongly deoxygenized environment. Under such conditions, regeneration of the passive layer of oxides becomes limited, particularly for 4H13 stainless steel, whose corrosion resistance relies on the continuous maintenance of a chromium oxide layer.^31^ In contrast to oxygen-containing systems, where oxygen depletion appears to occur only locally, nitrogen nanobubbles may reduce oxygen availability throughout the entire liquid volume.^28,32^ Consequently, corrosion is no longer confined to discrete anodic regions, the ratio of anode-to-cathode surface increases, and therefore the process can proceed more uniformly across the whole metal surface. The observation of uniform corrosion, even in chloride-free water, is consistent with this proposed interpretation.

Interestingly, the corrosion patterns observed in oxygen- and nitrogen-nanobubble dispersions may be interpreted within a common framework based on oxygen availability at the steel surface.^33^ Oxygen-containing systems appear to represent a regime of localized oxygen depletion, generating differential aeration cells, and promoting localized corrosion. In contrast, nitrogen nanobubbles create conditions approaching global oxygen depletion, limiting passive-film regeneration across the entire surface and resulting in more uniform corrosion. Thus, oxygen- and nitrogen-nanobubble dispersions may be considered two limiting cases of the same oxygen-availability-driven phenomenon, as schematically illustrated in Figure 3.

**Figure 3:**
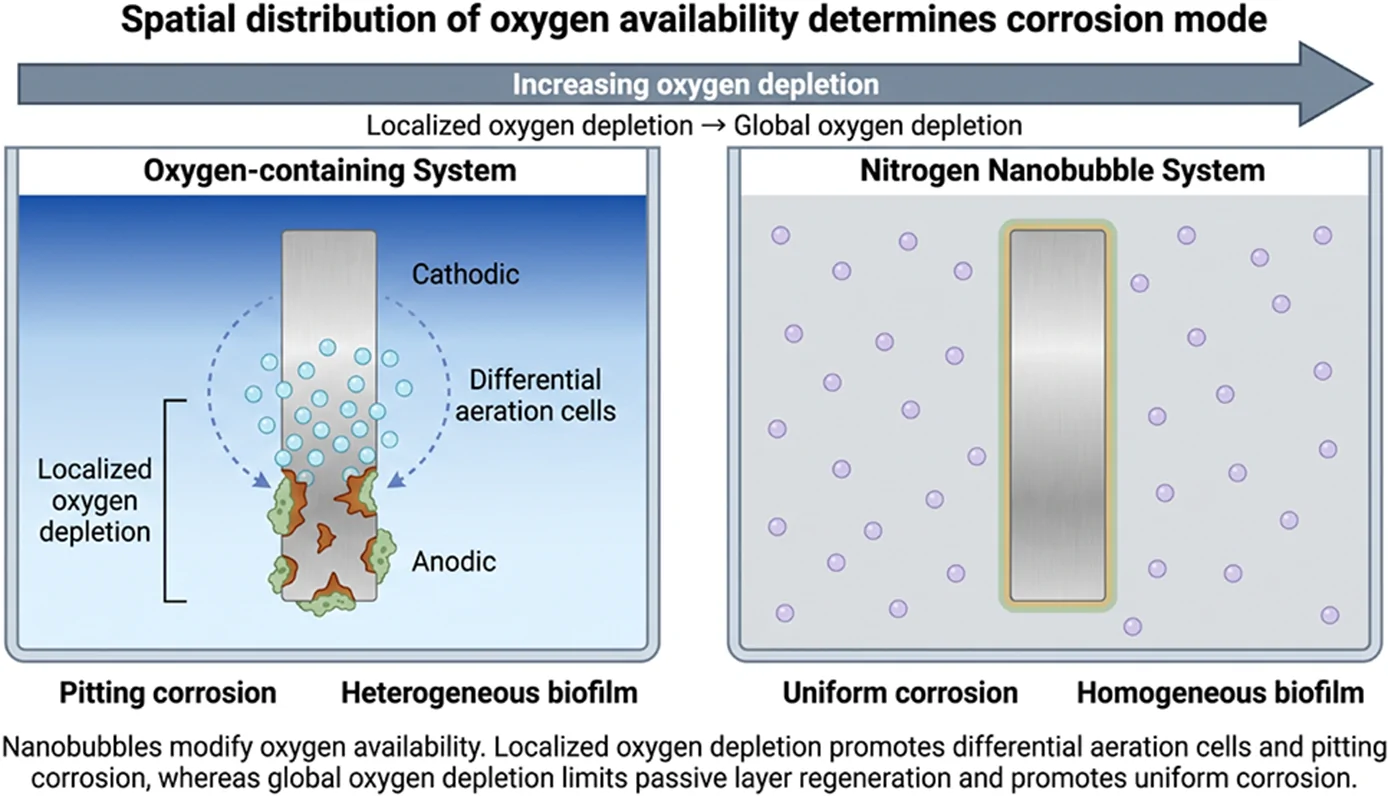
Proposed mechanism explaining the corrosion patterns observed for oxygen- and nitrogen-nanobubble dispersions. Localized oxygen depletion is proposed to generate differential aeration cells and localized corrosion, whereas near-complete oxygen depletion may limit passive film regeneration and promote uniform corrosion. Oxygen- and nitrogen-nanobubble dispersions are proposed to represent two limiting cases of the same oxygen-availability-driven phenomenon.

Taken together, these observations suggest that the corrosion mode is primarily governed by the spatial distribution of oxygen availability rather than by the direct chemical action of the gas within nanobubbles. Local oxygen gradients favor localized corrosion, whereas uniform oxygen depletion promotes uniform surface degradation. Direct verification of the proposed oxygen gradients and electrochemical processes will require dedicated measurements in future studies.

### 3.2 Corrosion Morphology Governs Bacterial Adhesion

The distinct corrosion morphologies observed after exposure to different nanobubble dispersions were directly reflected in the subsequent patterns of bacterial colonization, as visualized in Figure 4. Samples immersed in the reference and oxygen-nanodispersion-treated samples exhibit patchy coverage by bacteria (as visualized by crystal violet staining), while nitrogen-nanodispersion-treated ones display homogeneous coverage at both salinities.

**Figure 4:**
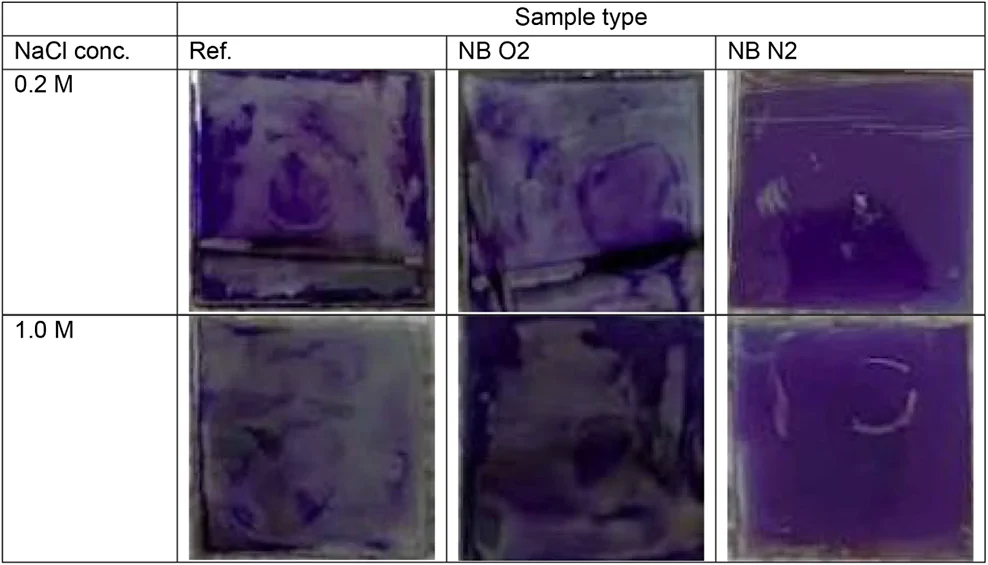
Exemplary macrophotographs of the steel coupons after crystal violet staining for 2 salinities, either 0.2 or 1.0 M, for all three types of samples.

Images from optical microscopy (upper row of Figure 5) confirmed localized colonization by bacteria on either reference or oxygen-nanobubble-enriched samples, which were focused around pits, cracks, and adhered corrosion products. On the other hand, for the nitrogen-nanobubble-treated ones, one can observe a homogeneous and dense layer of bacteria.

**Figure 5:**
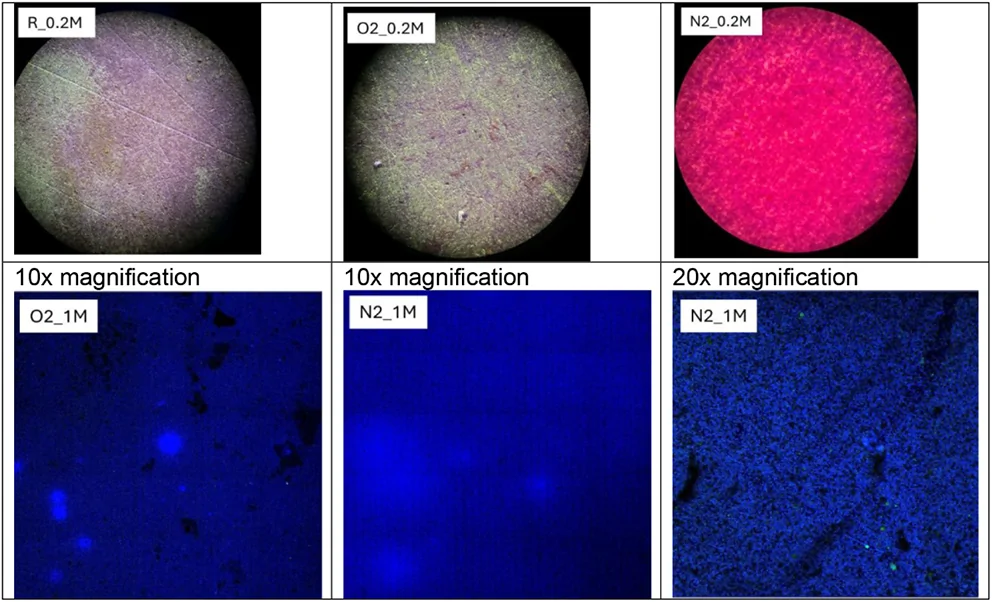
Exemplary optical microscopy (top row) and confocal microscopy (bottom row) photographs. All photos beside the bottom-right one are taken at 10× magnification, while for the nitrogen-nanobubble sample an additional magnification of 20× is presented.

Confocal microscopy with DAPI staining (bottom row of Figure 5) further confirmed the colonization patterns observed using crystal violet staining and, as a consequence, bacterial colonization. This confirms that crystal violet is adhered to bacteria and that the staining does not led to the adhesion to the steel itself. Higher magnification (bottom right image in Figure 5) shows the dense coverage of cells on the steel surface.

The observations suggest that bacterial colonization was governed primarily by the surface morphology generated during corrosion, similar to results presented in the literature.^34,35^ Localized corrosion produced isolated adhesion niches associated with pits and corrosion products, whereas uniform corrosion generated a rough surface that promoted a more homogeneous biofilm development.

Because bacterial colonization experiments were performed after completion of the corrosion exposure stage, bacteria were never directly exposed to nanobubble dispersions. Therefore, the observed differences in the biofilm architecture cannot be attributed to a direct interaction between nanobubbles and microorganisms. Instead, nanobubbles influenced bacterial adhesion indirectly by modifying oxygen availability, corrosion mode, and the resulting surface morphology.

## 4 Conclusions

This study demonstrates that gas-specific nanobubble dispersions control the corrosion mode of 4H13 martensitic stainless steel through modification of oxygen availability at the metal–liquid interface. Oxygen-containing systems were associated with localized oxygen depletion and differential aeration cells, resulting in nonuniform corrosion, whereas nitrogen nanobubbles generated near-global oxygen depletion and more uniform surface degradation. These distinct corrosion morphologies directly influenced bacterial colonization patterns, leading to either patchy or homogeneous biofilms. The results suggest that the spatial distribution of oxygen, rather than the direct chemical action of the gas enclosed in nanobubbles, governs the transition between localized and uniform corrosion. Consequently, nanobubbles may represent a simple tool for controlling both electrochemical surface processes and subsequent biological colonization.

## Supplementary Material



## Notes

Quantitative results for crystal violet in the presence of iron-rich corrosion products; additional images of bacterial colonization on steel; and optical microscopy images after 3 days of exposure to a corrosive environment (PDF)
