# Supplementary material for: Nanobubble-Induced Oxygen Gradients Control Corrosion Mode and Bacterial Adhesion on Martensitic Stainless Steel
Source: ACS Omega. 2026 Sep 29;11(40):62196–201. doi: 10.1021/acsomega.6c09261 (PMC13670875; doi:10.1021/acsomega.6c09261)
Supplement: Supplementary file 1 [file ao6c09261_si_001.pdf]

Nanobubble-induced oxygen gradients control corrosion mode and bacterial adhesion on martensitic stainless steel

Kacper Kołodziejski, Maja Goworek, Karol Ulatowski*, Rafał Podgórski, Paweł Sobieszuk

Warsaw University of Technology, Faculty of Chemical and Process Engineering, Department of Biotechnology and Bioprocess Engineering, Waryńskiego 1, 00-645 Warsaw, Poland

* corresponding author, e-mail: Karol.Ulatowski@pw.edu.pl

Keywords: nanobubbles, corrosion, bacterial adhesion, biofilm, oxygen gradients, stainless steel

Abstract

Biofilm formation is a large problem in corrosion prevention and can be part of a positive feedback loop, where a corroded surface is prone to bacterial adhesion, and bacteria themselves provide an environment that increases the corrosion rate of the metal surfaces. Nanobubbles are increasingly recognized as active modifiers of solid–liquid interfaces; however, their role in coupled corrosion and biofilm formation remains unclear. Here, we show that gas-specific nanobubble dispersions control both the corrosion mode of martensitic stainless steel (4H13) and the resulting Escherichia coli adhesion. Steel samples were immersed in oxygen or nitrogen nanobubble dispersions under varying salinity conditions up to 1.0 M NaCl. Oxygen nanobubbles induced spatially heterogeneous corrosion, characterized by localized pitting and differences along the sample height, while nitrogen nanobubbles resulted in uniform surface degradation, even in deionized water. These corrosion patterns directly translated into distinct biofilm structures: patchy biofilms formed on oxygen-treated surfaces, whereas nitrogen nanobubbles promoted a uniform biofilm. These observations are consistent with nanobubble-driven oxygen transport at the interface. We propose that oxygen concentration gradient along the length of the steel coupon promotes differential aeration cells and pitting corrosion, whereas nitrogen nanobubbles deoxygenate the system, destabilizing the passive layer and leading to uniform corrosion. Consistent qualitative observations from both crystal-violet and DAPI staining reveal a strong coupling between nanobubble-mediated oxygen gradients, corrosion morphology, and microbial colonization. These findings highlight nanobubbles as a simple tool for controlling interfacial electrochemical processes and biofilm formation.

Supporting information

S1. Quantitative results of crystal violet in presence of iron-rich corrosion products

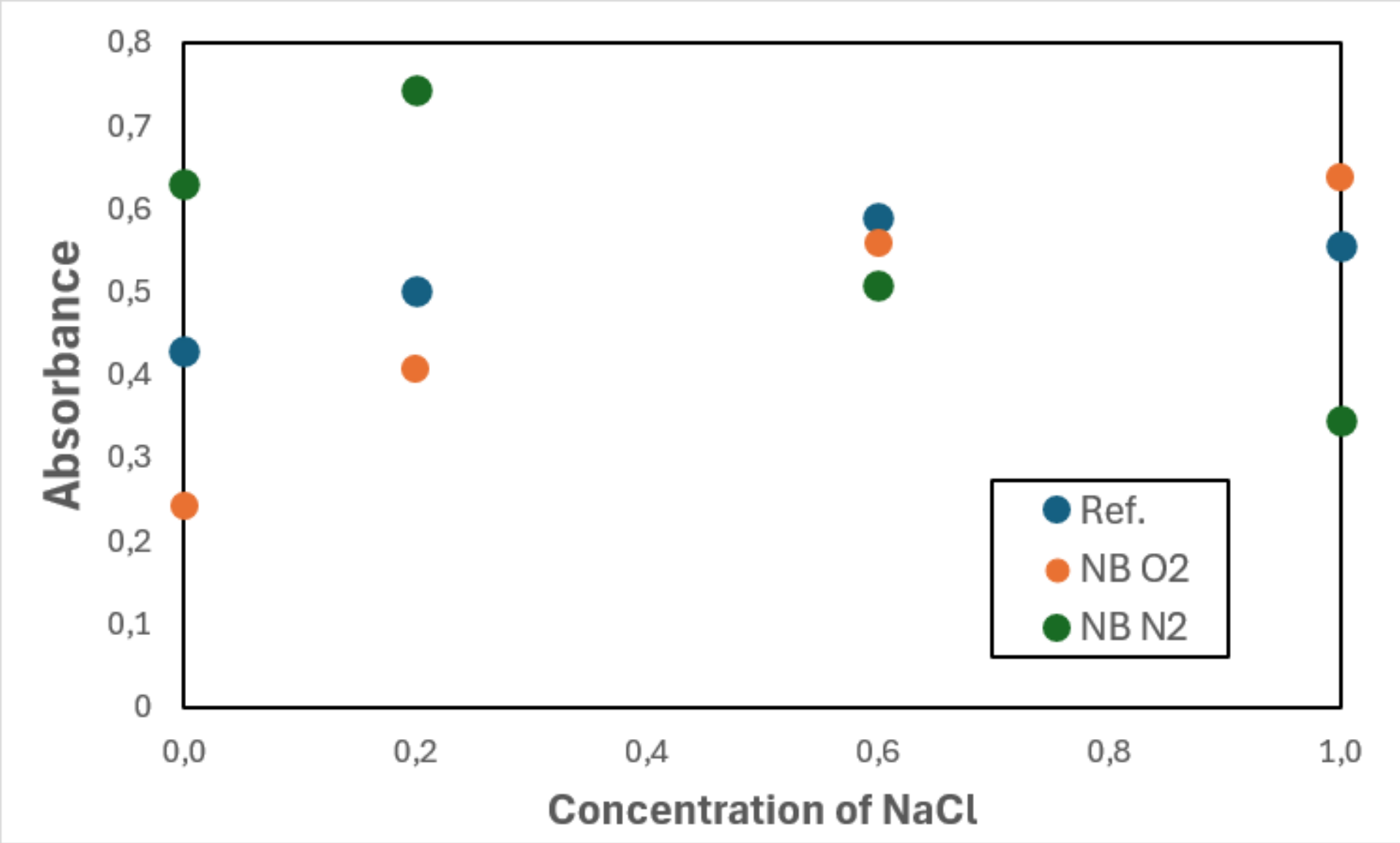

Figure S1. Quantitative crystal violet assay results in form of scatter plot.

Table S1. Quantitative crystal assay results with standard deviation in tabular form

Concentration of NaCl	0.0	0.2	0.6	1.0
**Ref.**	0.428±0.058	0.500±0.159	0.590±0.147	0.556±0.270
**NB O2**	0.243±0.105	0.407±0.161	0.559±0.189	0.637±0.050
**NB N2**	0.631±0.082	0.744±0.180	0.509±0.084	0.345±0.210

Qualitative crystal-violet staining suggested trends are not consistent with microscopic observations. However, quantitative interpretation by crystal violet was complicated by the presence of corrosion products. During the acetic-acid destaining step, iron-containing deposits are partially dissolved (as described in literature [32]), potentially contributing to absorbance measurements. Consequently, the conclusions of the present study are primarily based on consistent macroscopic and microscopic observations.

S2. Complete set of sample photographs

	Sample type		
NaCl conc.	Ref.	NB O2	NB N2
0.0 M	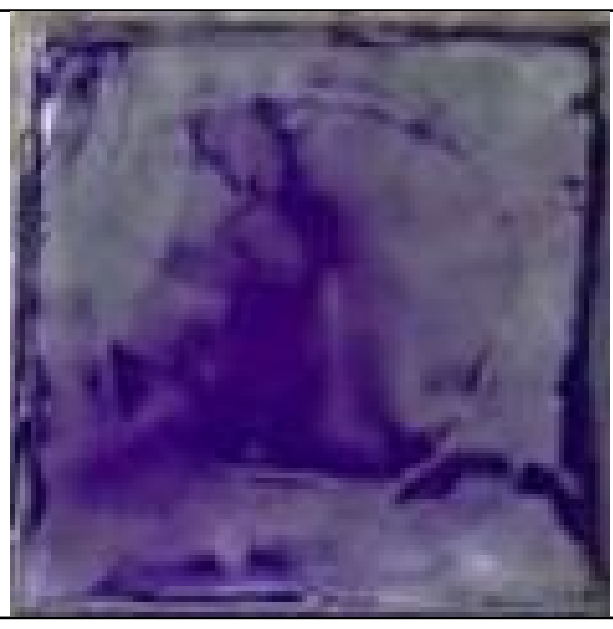	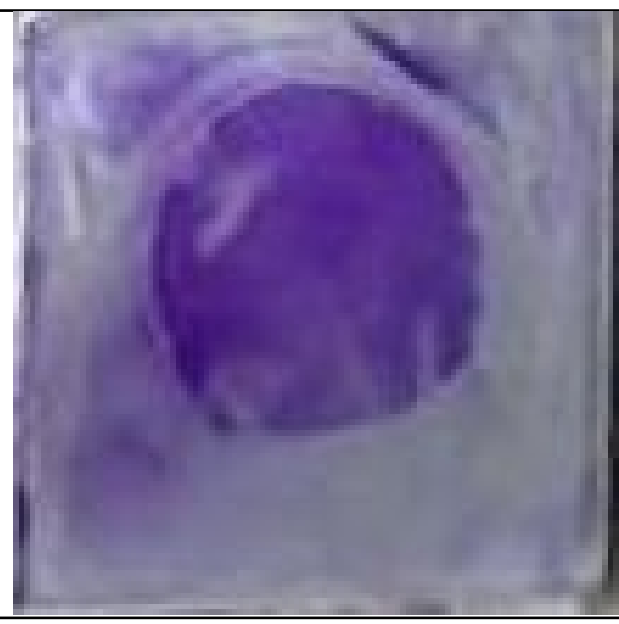	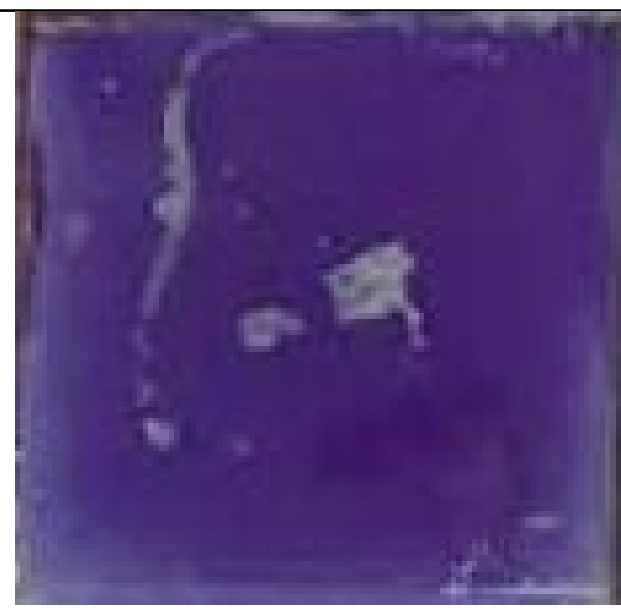

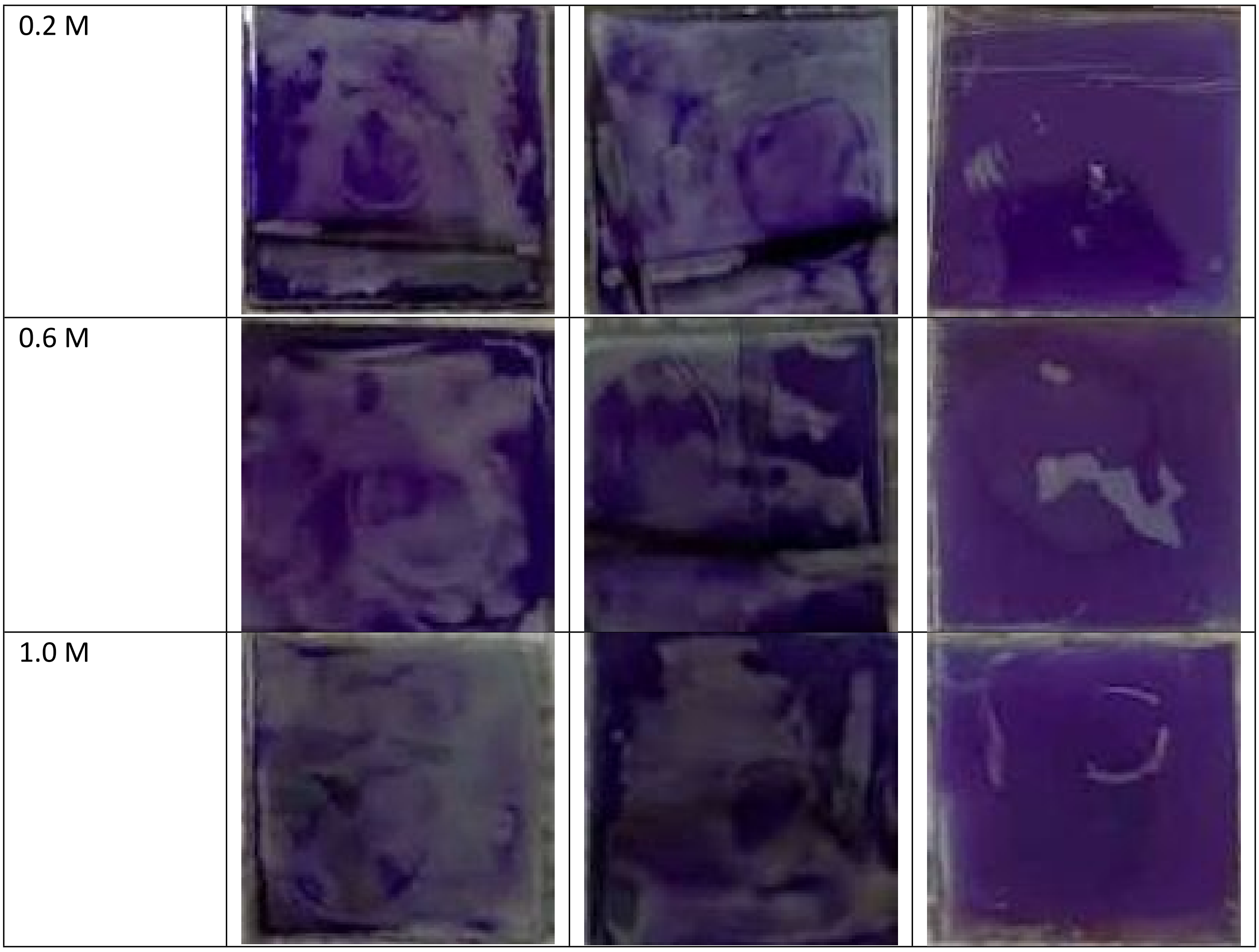

Figure S2. Macrophotographs of steel samples after bacterial colonisation and crystal violet staining for reference, oxygen- and nitrogen-nanobubble-enriched salines

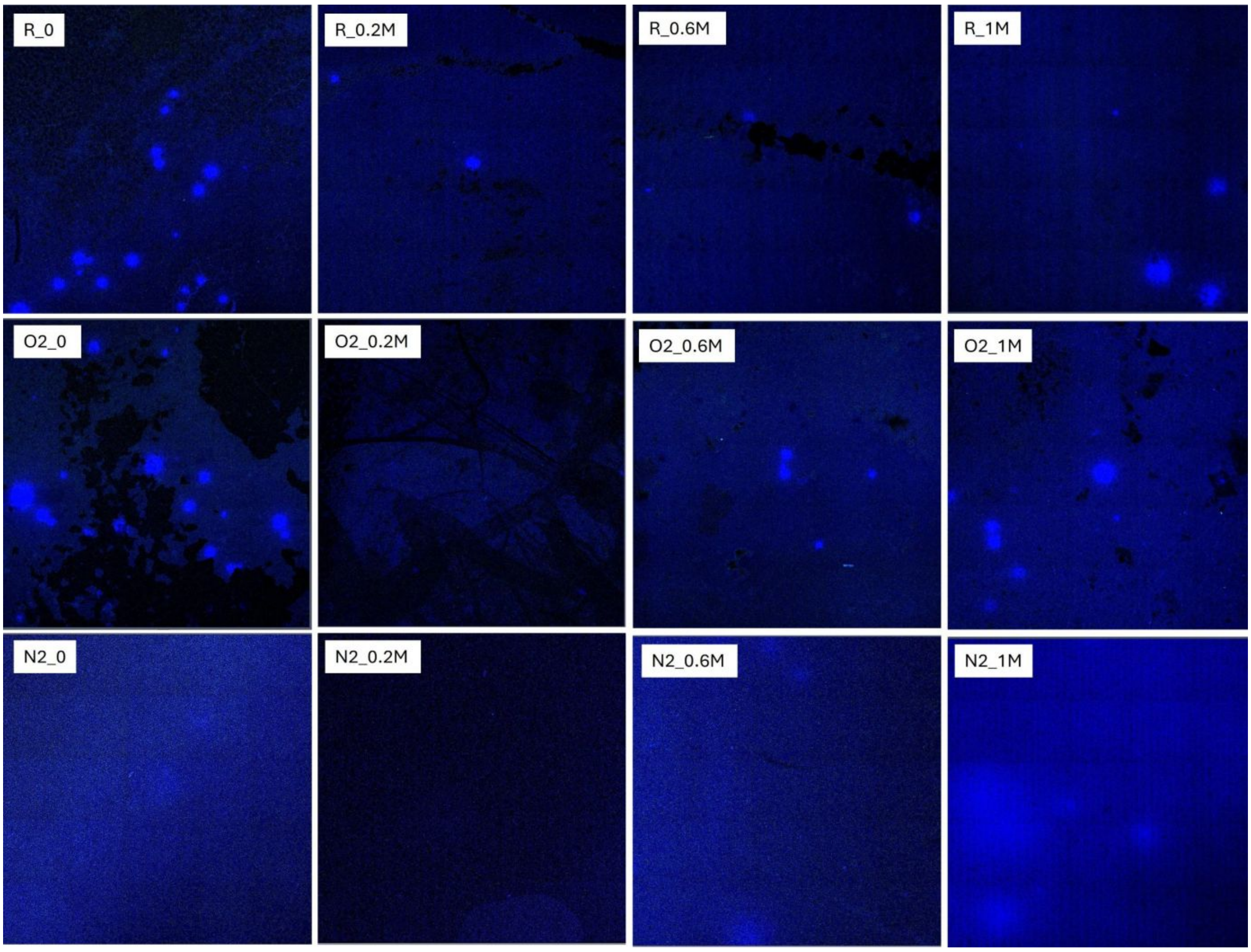

Figure S3. Confocal microscopy photographs (10x magnification) of steel samples after bacterial colonisation and DAPI staining for reference, oxygen- and nitrogen-nanobubble-enriched salines

S3. Set of optical microscopy photographs taken after 3 days of exposure to corrosive environment

**Sample type**	**Concentration of sodium chloride**			
	**0.0 M**	**0.2 M**	**0.6 M**	**1.0 M**
**Ref.**	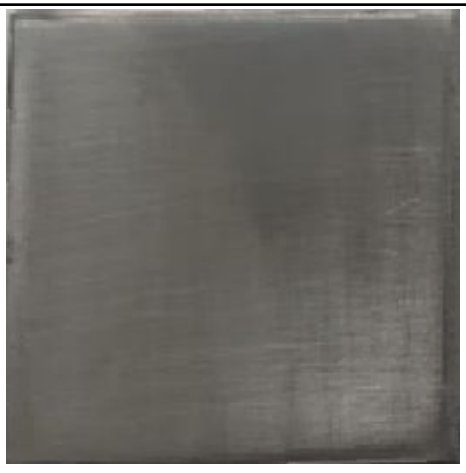	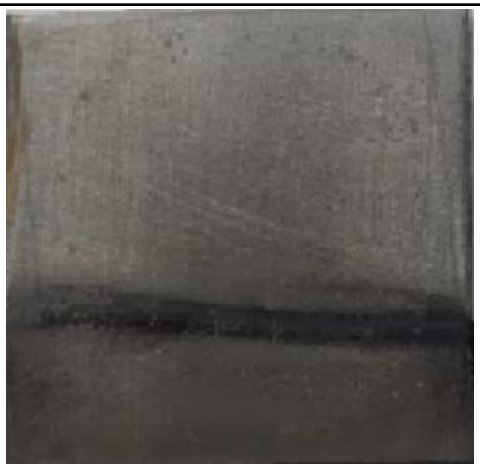	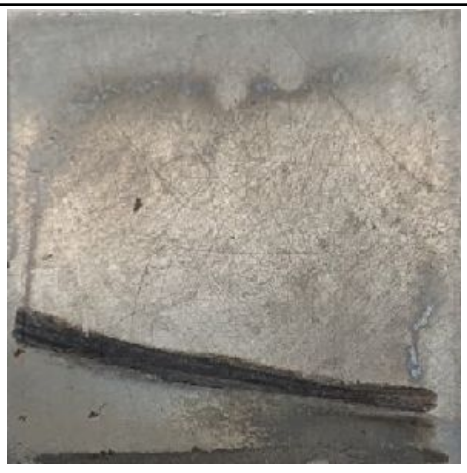	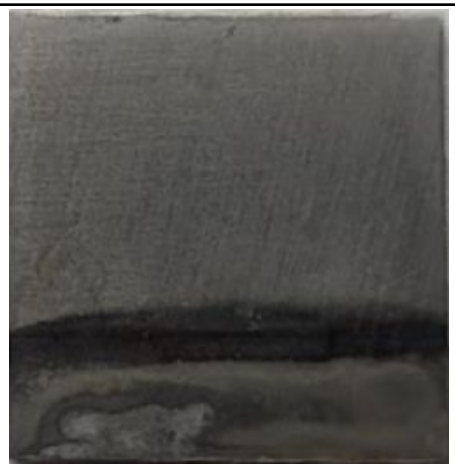
**NB O2**	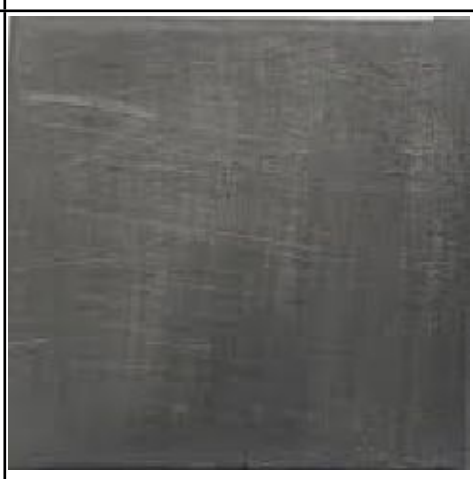	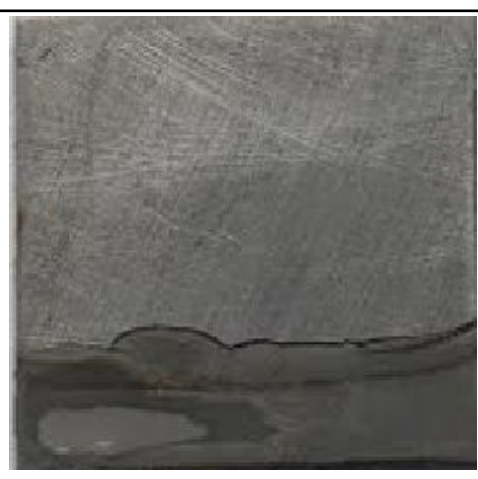	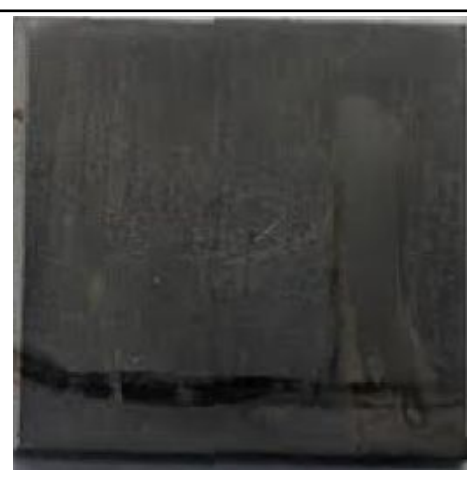	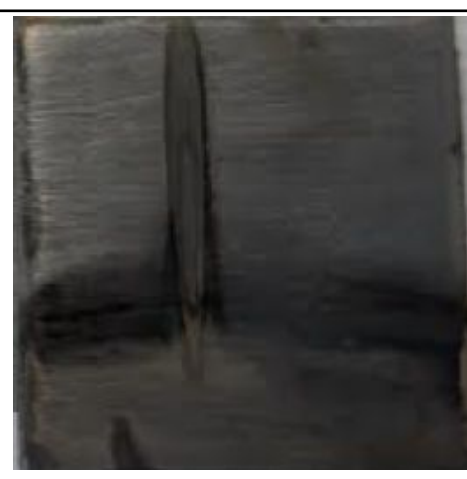
**NB N2**	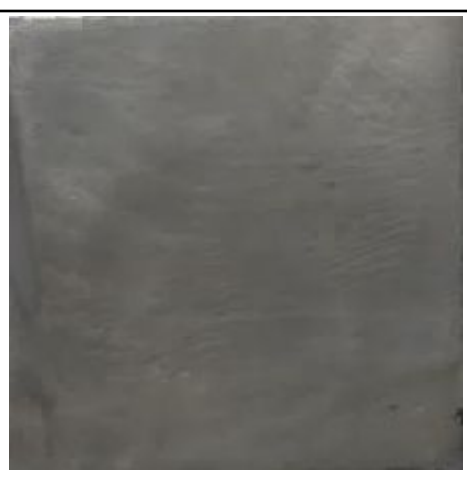	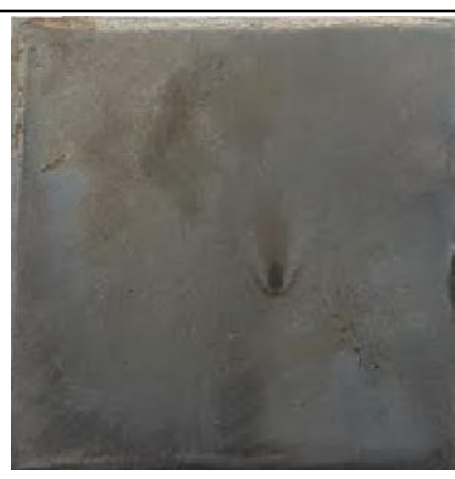	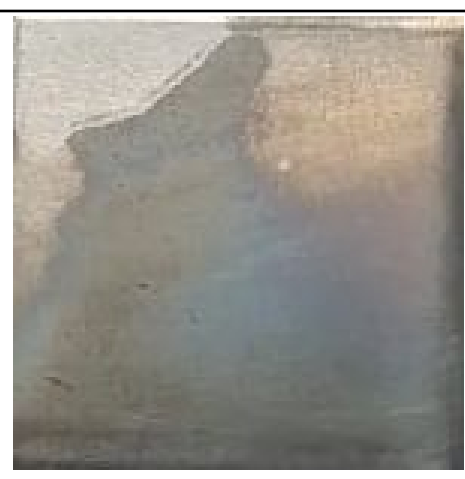	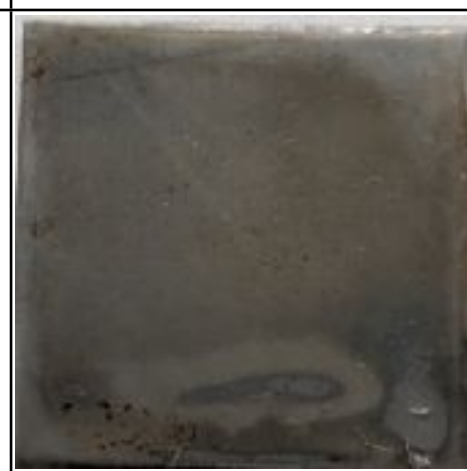

Figure S4. 4H13 steel plates after 3-day exposure to the corrosive environment without nanobubbles dispersed in the NaCl solution (denoted as “Ref.”) or with either oxygen nanobubbles (denoted as “NB O2”) or nitrogen nanobubbles (denoted as “NB N2”) dispersed.
